# Trajectories of social class and adult self‐perceived oral health

**DOI:** 10.1111/cdoe.13001

**Published:** 2024-08-15

**Authors:** Reem Aljubair, Elsa Karina Delgado‐Angulo

**Affiliations:** ^1^ Dental Public Health Group, Faculty of Dentistry, Oral & Craniofacial Sciences King's College London London UK; ^2^ Facultad de Estomatología Universidad Peruana Cayetano Heredia Lima Peru

**Keywords:** life course perspective, oral health, social class, social mobility

## Abstract

**Objectives:**

To determine the effect of social mobility on self‐perceived oral health (SPOH) by: (i)characterizing patterns of social mobility from birth to adulthood and (ii)assessing their influence on SPOH among British adults.

**Methods:**

A secondary data analysis of the 1970 British Cohort Study. Data were collected at birth and at 5, 10, 16, 26, 30, 34, 38, 42 and 46 years of age. Social class (SC) was indicated by parental SC from birth to age 16 and own SC from ages 26 to 42. At age 46, SPOH was measured using a single question. Sex, ethnicity, country and residence area were included as potential confounders. Latent class growth analysis (LCGA) was used to identify trajectories of exposure to non‐manual SC over time, instead of predetermined categories.

**Results:**

LCGA identified four social mobility patterns: stable high, stable low, upwardly mobile and downwardly mobile; the time for the change in SC happening between 16 and 26 years. A total of 9657 participants were included. In the crude model, stable high had lower odds (OR: 0.67, 95% CI: 0.59–0.76), while downward mobility and stable low had higher odds (OR: 1.36, 95% CI: 1.15–1.61 and OR: 1.57, 95% CI: 1.40–1.77) of poor SPOH than upward mobility. These results were corroborated in the fully adjusted model; being female and living in rural areas was also associated with lower odds (OR: 0.64, 95% CI: 0.59–0.71 and OR: 0.90, 95%CI: 0.80–1.00) of poor SPOH.

**Conclusion:**

Social mobility significantly affects SPOH in British adults. Those in non‐manual SC have better SPOH than those in manual SC. When compared to upward mobility, downwardly mobile individuals report bad SPOH more frequently, evidencing that current SC influences oral health in a slightly greater measure than early years SC.

## 1 INTRODUCTION

A life course approach recognizes the importance of time (and timing) in understanding processes that influence morbidity and mortality; and to clarify them, three conceptual models have been proposed, namely: critical periods, accumulation, and social trajectories. The critical period model emphasizes the timing of exposure; the accumulation model pays attention to the amount—or duration—of exposure; and the social trajectories or pathways model, focuses on the sequence of exposure.^1^, ^2^ Trajectories refer to chains of risk in which one exposure tends to lead to another and then another and different chains, or trajectories, can lead to increased or decreased risk.^2^ All three models could be applied to the field of dentistry; however, the social trajectories model has shown to be the most appropriate to describe the association between social class and adult oral health.^3^

Social mobility refers to the movement of individuals or families across different social classes typically as a result of changes in education, occupation, income, or other socioeconomic factors.^4^ Social mobility is considered good for society because it encourages placement of individuals in social positions according to competence rather than their social origin^5^ and is viewed as a social policy to reduce health inequality.^4^, ^6^

Previous research has shown that changes in social mobility can significantly affect a person's physical and psychological well‐being, including various oral health outcomes.^7^, ^8^, ^9^, ^10^, ^11^, ^12^, ^13^ In the Dunedin Study, upward mobility was associated with better dental but not periodontal status while downward mobility was associated with worse dental but not periodontal status.^7^, ^9^ Pearce et al.,^8^ using the Newcastle Thousand Families cohort, found that social mobility was linked to the retention of functional oral health until age 50, particularly among women. Bernabe et al.^10^ in Finland, found a gradient in periodontal disease, edentulism and dental caries among different social mobility groups, although differences were only found for edentulism and dental caries. In the context of the 1982 Pelotas birth cohort, Peres et al.^11^ found that adolescents experiencing persistent poverty had higher levels of decay and unmet treatment needs compared to those who never experienced poverty. In Australia, individuals in the stable social disadvantage group had more oral impacts than the reference category of upwardly mobile persons.^12^ Moreover, a recent systematic review concluded that belonging to a persistently low SES throughout life increases the chances of tooth loss and that low SES in adulthood and downward mobility have stronger association with tooth loss than low SES in childhood, although the latter needs to be corroborated with further studies.^13^

While these studies provide valuable insights into the relationship between social mobility and oral health, they do not capture the full impact of mobility accurately as they derive four social trajectories (always low, upwardly mobile, downwardly mobile and always high) from two waves of socioeconomic data (childhood and adulthood), the simplest scenario in life course epidemiology, and as such, unlikely to represent the entire array of social circumstances that individuals experience across their lifespan.

To address the limitation of previous studies, latent class growth analysis (LCGA) is proposed as a valuable alternative in this study as it allows the classification of individuals into distinct groups with similar developmental trajectories, revealing underlying patterns and variations that may not be evident through traditional analytical approaches.^14^, ^15^ Previous studies utilizing LCA in this context have been limited to measuring the outcome of social mobility only until early 30s, neglecting the long‐term effects of social mobility on oral health in adulthood.^16^, ^17^

Given these gaps in knowledge, the present study utilizes the longitudinal data from the British Cohort Study (BCS70) and applies LCGA to examine the relationship between social mobility patterns and self‐perceived oral health (SPOH). The aim of this study was to determine the effect of social mobility on SPOH through two specific objectives: (i) to characterize changes in social class (social mobility) from birth to adulthood and (ii) to assess the influence of these changes in social class on SPOH in British adults.

## 2 METHODS

Data for this study came from the 1970 British Cohort Study (BCS70), a longitudinal study following individuals born in Great Britain during a specific week in 1970, with a baseline response rate (RR) of 96%. The 628 participants born in Northern Ireland were excluded from subsequent waves; but, immigrants born in the reference week were added to the study when cohort members (CMs) were traced through schools.^18^ Data collection involved questionnaires as well as extraction of relevant information from clinical records to gather data on physical, educational and social development, economic circumstances and health among other factors over the course of CM's lives. The follow‐up data were collected at age 5 (RR = 77%), 10 (RR = 86%), 16 (RR = 67%), 26 (RR = 52%), 30 (RR = 66%), 34 (RR = 56%), 38 (RR = 52%) and 42 (RR = 57%) years with the latest sweep happening when participants were 46–48 years old (RR ~ 52%); the exact number of expected and achieved sample per wave has been reported previously.^19^, ^20^ Due to selective attrition, the sample included a greater proportion of CM from a higher childhood socioeconomic background and females.^18^, ^19^, ^20^

SPOH, the outcome, was assessed at age 46 using a five‐point scale (excellent, very good, good, fair and poor) answering the question “Did you consider your dental health (including your mouth, teeth, and/or dentures) to be …”. These five categories were collapsed into two, namely Good (including excellent, very good and good answers) and Poor (fair and poor).

Social mobility patterns were the primary exposure, determined by parental social class at birth, age 5, 10 and 16 and by own occupation social class at ages 26, 30, 34, 38 and 42, using the Registrar General's six‐group classification of occupations, namely professional (I), managerial and technical (II), skilled non‐manual (IIINM), skilled manual (IIIM), partly skilled (IV) and unskilled (V).^21^ In case of missing information or unemployment of the paternal figure, the SC of the mother was used as proxy; a period of unemployment of the CM yielded on missing information for that wave. Binary indicators were created at each time point by collapsing the six classes into: 0 for non‐manual (classes I, II and IIINM) and 1 for manual (classes IIIM, IV and V). LCGA, a robust method of clustering,^22^ was used to determine different trajectories of exposure to non‐manual social class. LCGA is a special type of growth mixture model characterized by having zero variances and covariances treating individuals within a class as homogeneous with respect to their development.^14^ LCGA generates fit statistics and is useful in finding cut points on the growth factors,^14^ determining the most appropriate number of clusters for a population. Missing data are handled as if the probability of being missing is the same only within groups defined by the observed data, the data are considered missing at random (MAR). Because of this, the use of several different sets of starting values is recommended.^14^ This process is automated and LCGA was conducted for the whole sample. The final number of trajectories was determined using the Bayesian information criterion (BIC) and sample size adjusted BIC^23^; the goal being to identify the smallest number of trajectories that best fit the data. Then the probability of each participant belonging to each of the different latent classes was estimated and a new categorical variable was created representing such assignment. This indicator variable was included as a predictor in the regression models for SPOH.

Participants' sex, ethnicity (White British vs. others), country within the United Kingdom dichotomised as England and others (Wales and Scotland) and residence area (urban vs. rural) were included as confounders. Demographic characteristics, such as sex and ethnicity, are considered important social determinants of health and have been linked to various oral health outcomes including perceived oral health^24^, ^25^, ^26^, ^27^, ^28^ and could affect how social mobility relates to oral health.^29^ The same can be said about area characteristics, such as country and area of residence, as variations in healthcare access and oral health behaviours may influence the perception of own's oral health status^26^, ^27^, ^28^, ^30^ and the association between social mobility and oral health outcomes.^31^ On the other hand, there are social mobility differences across the United Kingdom; social mobility not only depend on origins, education and skills, but also country and area of residence.^32^ There are also marked differences in social mobility between ethnic group, with some groups receiving better education than others yet not always better occupational outcomes.^32^ Important gaps are also found in relation to sex, as women are less likely than men to be in higher professional occupations.^32^

Logistic regression models were fitted and odds ratios (OR) were reported to assess the relationship between social mobility group assignment and lifetime SPOH in crude and adjusted models using the best social mobility scenario as the reference category. Additionally, a second set of regression models were fitted using the upwardly mobile category as reference to elucidate if proximal or distal exposure to manual social class have a greater effect on SPOH.

## 3 RESULTS

### 3.1 Changes in social class

**FIGURE 1:**
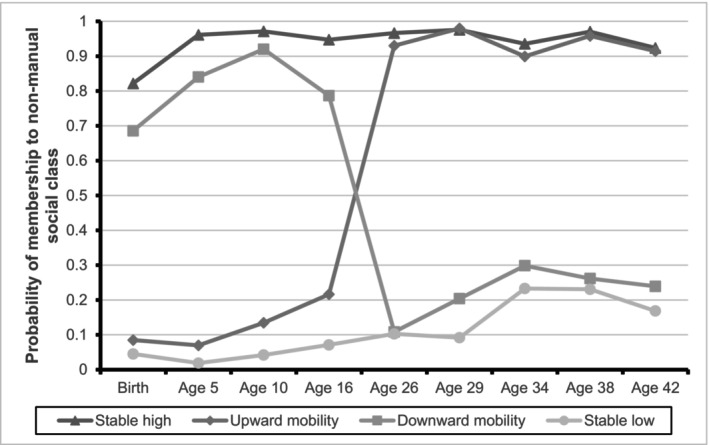
Probability of membership to non‐manual social class in four social class trajectories from birth to age 42.

The LCGA involved all 21, 612 individuals who had information on social class on at least one time point. The model selection process involved evaluating the Bayesian Information Criterion (BIC) and adjusted BIC values; with the four‐trajectory model providing the best fit for the BCS70 data. Solutions with more classes showed minimal decreases in the BIC and adjusted BIC values, as indicated in Table 1. The four patterns identified based on the probability of membership in the non‐manual social class (Figure 1) were as follows: (i) Stable high: individuals who were in the non‐manual social class throughout the study period, it accounted for 28% of the LCGA sample; (ii) Stable low: individuals who were persistently in the manual social class over time; it represented 28% of the sample; (iii) Upward social mobility: Individuals who were in the manual social class during childhood but moved to the non‐manual social class at age 16 and remained on it, accounting for 38% of the sample; and (iv) Downward social mobility: individuals who left the non‐manual social class after the age of 16 and remained in the manual social class, it represented 6% of the population.

**TABLE 1:** Model‐fit indices for alternative latent class analyses for social class trajectories.

Model‐fit indices	Number of classes		
	Two	Three	Four
Number of parameters	19	29	39
Log likelihood	−53329.78	−49320.87	−48195.48
BIC	106 849	98 930.86	96 779.76
% change in BIC		−7.04%	−2.17%

Abbreviation: BIC, Bayesian information criterion.

### 3.2 Description of the sample

Of the initial 21 612 individuals, only 10 645 had information on SPOH and among them 130 did not have information on their ethnic group, yielding a sample size of 10 515 participants. A further 858 participants were dropped from the analytical sample because of incomplete information on their social class during childhood or adulthood, hence the final analytical sample consisted of 9657 individuals (45% of the initial population). Of them, 48% were men and 52% women, over 97% of the sample identified as White British with a 87% of the participants residing in England and 70% living in urban areas. Furthermore, in terms of social mobility, 32% belonged to the stable high group, 33% were upwardly mobile, 9% downwardly mobile and 26% were in the stable low group.

In Table 2, statistically significant differences in the proportion of participants with good and poor SPOH according to their social class at different waves could be observed (*p* < .001). In all, there is a greater proportion of participants rating their oral health as good among the non‐manual social class as compared to those in manual social class at every point in time both among males and females.

**TABLE 2:** Self‐perceived oral health (SPOH) in men and women by social class at time points (*n* = 9657).

Social class	Men				Women			
	*n*	%	Good SPOH	Poor SPOH	*n*	%	Good SPOH	Poor SPOH
At birth								
Non‐manual	1584	36.9	74.7	25.3	1170	37.6	83.0	17.0
Manual	2704	63.1	67.2	32.8	2935	62.4	78.0	22.0
At age 5								
Non‐manual	1528	40.9	75.8	24.2	1645	40.2	84.8	15.2
Manual	2209	59.1	65.7	34.3	2442	59.8	77.0	23.0
At age 10								
Non‐manual	1789	44.9	74.5	25.5	1975	45.4	85.0	15.0
Manual	2191	55.1	65.8	34.2	2374	54.6	76.2	23.8
At age 16^a^								
Non‐manual	1178	51.3	75.6	24.4	1456	51.9	86.1	13.9
Manual	1119	48.7	69.4	30.6	1349	48.1	80.8	19.2
At age 26^b^								
Non‐manual	1436	57.2	79.4	20.6	2348	79.8	82.9	17.2
Manual	1073	42.8	67.4	32.6	594	20.2	76.3	23.7
At age 30								
Non‐manual	2217	59.6	75.7	24.3	2889	80.7	83.8	16.2
Manual	1501	40.4	64.5	35.5	690	19.3	75.7	24.3
At age 34^c^								
Non‐manual	2128	61.1	77.1	22.9	2646	80.2	83.3	16.7
Manual	1355	38.9	65.4	34.6	652	19.8	74.7	25.3
At age 38								
Non‐manual	2216	65.3	76.4	23.6	2642	80.0	83.1	16.9
Manual	1176	34.7	66.6	33.4	659	20.0	76.8	23.2
At age 42								
Non‐manual	1035	59.5	73.0	27.0	1493	70.3	81.3	18.7
Manual	704	40.5	53.1	46.9	630	29.7	69.8	30.2

^a^ Response handicapped by teachers' strike.^19^

^b^ Response handicapped by use of postal survey and limited time and funds available for tracing.^19^

^c^ Target sample reduced to meet funding limitations from this wave on.^19^

There were statistically significant associations between the confounders and both the main exposure and outcome of this study, as presented in Tables S1 and S2.

### 3.3 Association between social mobility and SPOH

The results from the logistic regression modelling showed, unsurprisingly, that any social mobility that involved a period in manual social class were more likely to report poor SPOH when compared to those in the stable high group (Table 3A). When using the upward social mobility as the reference category to try to elucidate if there were any differences between moving downwardly of upwardly in the social scale (Table 3B), the unadjusted model showed that individuals in the stable high group displayed significantly lower odds (OR: 0.67, 95% CI: 0.59–0.76) of reporting poor SPOH compared to those in the reference category. Conversely, individuals in the downward social mobility and stable low groups exhibited significantly higher odds (OR: 1.50, 95% CI: 1.27–1.76 and OR: 1.74, 95% CI: 1.55–1.96, respectively) of reporting poor SPOH than those in the upward social mobility group. The fully adjusted model revealed similar results; individuals in the stable high group retained significantly lower odds (OR: 0.65, 95% CI: 0.57–0.74) of poor SPOH, and individuals in the downward social mobility and stable low groups exhibited significantly higher odds of poor SPOH than the reference category (OR: 1.36, 95% CI: 1.15–1.61 and OR: 1.57, 95% CI: 1.40–1.77, respectively) when taking into account the effects of gender, ethnicity, country and area of residence (Table 3). In addition, females and participants living in rural areas had lower odds (OR: 0.64, 95% CI: 0.59–0.71 and OR: 0.90, 95%CI: 0.80–1.00, respectively) of poor SPOH than their counterparts.

**TABLE 3:** Association between social class trajectories and self‐perceived oral health (SPOH) at 46–48 years of age using the stable low (3A) and the upward mobility (3B) trajectories as reference (*n* = 9657).

	Unadjusted model		Fully adjusted model	
	OR	95% CI	OR	95% CI
3A				
*Social class trajectories*				
Stable high (*n* = 3049)	1.00	[Reference]	1.00	[Reference]
Upward mobility (*n* = 3221)	1.49	[1.32; 1.69]***	1.54	[1.36; 1.75]***
Downward mobility (*n* = 902)	2.23	[1.89; 2.65]***	2.10	[1.77; 2.49]***
Stable low (*n* = 2485)	2.60	[2.29; 2.94]***	2.43	[2.14; 2.75]***
Sex				
Male (*n* = 4633)			1.00	[Reference]
Female (*n* = 5024)			0.64	[0.59; 0.71]***
Ethnicity				
White British (*n* = 9373)			1.00	[Reference]
Other (*n* = 284)			0.94	[0.71; 1.25]
Country of residence				
England (*n* = 8380)			1.00	[Reference]
Others (*n* = 1277)			1.13	[0.99; 1.30]
Residence area				
Urban (*n* = 6791)			1.00	[Reference]
Rural (*n* = 2866)			0.90	[0.80; 1.00]*

3B				
*Social class trajectories*				
Upward mobility (*n* = 3221)	1.00	[Reference]	1.00	[Reference]
Stable high (*n* = 3049)	0.67	[0.59; 0.76]***	0.65	[0.57; 0.74]***
Downward mobility (*n* = 902)	1.50	[1.27; 1.76]***	1.36	[1.15; 1.61]***
Stable low (*n* = 2485)	1.74	[1.55; 1.96]***	1.57	[1.40; 1.77]***
Sex				
Male (*n* = 4633)			1.00	[Reference]
Female (*n* = 5024)			0.64	[0.59; 0.71]***
Ethnicity				
White British (*n* = 9373)			1.00	[Reference]
Other (*n* = 284)			0.94	[0.71; 1.25]
Country of residence				
England (*n* = 8380)			1.00	[Reference]
Others (*n* = 1277)			1.13	[0.99; 1.30]**
Residence area				
Urban (*n* = 6791)			1.00	[Reference]
Rural (*n* = 2866)			0.90	[0.80; 1.00]*

Abbreviations: CI, confidence interval; OR, odds ratio.

* *p <* .05.

^**^ *p <* .01.

^***^ *p <* .001.

Given the higher proportion of females on the analytical sample, a stratified analysis was conducted yielding the same trends as within the whole sample, albeit more pronounced among women (Table S3).

## 4 DISCUSSION

This study used nine waves of data on social class (from birth to age 42) from the BCS70 to identify common patterns of changes in social class using LCGA and explore how these trajectories were related to subjective oral health at age 46. Four distinctive patterns of social mobility were identified, corresponding to stable low, upward mobility, downward mobility and stable high groups, which led to differences in SPOH in adult life. There was a clear gradient in the prevalence of poor SPOH in terms of changes in social class. Individuals in the upward social mobility group were 54% more likely to report poor SPOH and those in downward social mobility and stable low groups (worst possible scenario) had 2.10 and 2.43 times the odds of poor SPOH compared to those in the stable high category. Furthermore, those in the downward social mobility group were 36% more likely to consider their OH poor when compared to the upward mobility category.

As for the first objective, the four trajectories identified with the help of LCGA coincide with the empirical trajectories consistently characterized in other birth cohorts with dental data.^8^, ^9^, ^11^ Furthermore, this analysis allowed the identification of the critical point at which social class changes, either to improve (upward mobility) or to decline (downward mobility). This moment seems to occur when individuals leave the parental home and become financially independent; in the case of this cohort, this happens between the ages of 16 and 26.

With regards to the second objective, social mobility was associated with SPOH in adults, and there was a clear gradient showing worse results among as the social mobility groups worsen. In addition, individuals in the downward social mobility group reported worse SPOH by age 46 years than those in the upward social mobility category, which is in line with previous research reporting that oral health in adult life may be more influenced by current rather than past socioeconomic experiences.^3^, ^10^, ^12^ Nonetheless, findings also suggest that living in manual social class early in life has a negative impact in SPOH, highlighting the importance of the early years in health.^33^, ^34^

While this research underscores a robust association between social mobility and subjective oral health, some limitations must be considered when interpreting its findings. First, is the use of occupation‐based social class to measure participants' socioeconomic status at different stages in life, disregarding other socioeconomic indicators relevant to oral health. Nonetheless, a key strength of the Registrar's General Social Class system is its past official status in the United Kingdom, and hence, its widespread use in vital statistics and censuses over a long period of time, and more importantly, it has been widely used to describe the socioeconomic gradient in health.^21^ In this same respect, LCGA was conducted among the whole sample instead of only those with information on social class for all waves and the statistical software deals with missing data automatically which could have introduced some bias; however, it has been shown using the BCS70 that the application of multiple imputation improves the precision on all variable estimates.^20^ Second, the study focused on a specific cohort born in Great Britain in 1970, which may restrict the generalizability of findings to other populations. Because of that, findings require replication in other cohorts in the United Kingdom and abroad, using alternative socioeconomic and oral health measures. Third, as the aim was to assess the overall impact of social mobility on adult SPOH rather than to study possible underlying mechanisms, analysis was descriptive and adjusted for confounders only; further studies could explore potential pathways for the association of social mobility to oral health. Fourth, the outcome measure for this study was the perception of own's oral health and, as such, self‐reported and prone to bias to some extent; hence, these results should be interpreted with caution as they do not necessarily reflect the clinical oral health status of participants.

In conclusion, this research sheds light on the complex interplay between social mobility, early‐life circumstances and oral health. It identified four groups with different mobility paths and highlighted a critical period when individuals become financially independent in which social circumstances could change permanently. Those in stable low, downward social mobility and upward social mobility had worse SPOH than the stable high social mobility group; downwardly mobile individuals reported worse SPOH than those in the upward social mobility group. These findings suggest that proximal social experiences might be more relevant to adult oral health than those in early life, although early exposure to disadvantageous conditions also plays a role in poor perception of own's oral health.

## CONFLICT OF INTEREST STATEMENT

The authors declare no conflict of interest in relation to this study.

## Supporting information


Table S1.
Table S2.
Table S3.

## Data Availability

The data that support the findings of this study are openly available in UK Data Archive at https://ukdataservice.ac.uk, reference number 200001.
