# Supplementary material for: Trajectories of social class and adult self‐perceived oral health
Source: Community Dent Oral Epidemiol. 2024 Aug 15;53(1):26–32. doi: 10.1111/cdoe.13001 (PMC11754143; doi:10.1111/cdoe.13001)
Supplement: Supplementary file 1 — Table S1. Table S2. Table S3. [file CDOE-53-26-s001.docx]

**Table S1. Distribution of the sample according to confounding variables across self-perceived oral health groups**

|  |  | **Self-perceived oral health** | | | |  |
| --- | --- | --- | --- | --- | --- | --- |
|  |  | ***Good*** | | ***Bad*** | | **p-value** |
|  |  | n | % | n | % |  |
| ***Sex*** | |  |  |  |  | <0.001 |
|  | Male | 3237 | 68.9 | 1396 | 30.1 |  |
|  | Female | 4004 | 79.7 | 1020 | 20.3 |  |
| ***Ethnicity*** | |  |  |  |  | 0.573 |
|  | White British | 7024 | 74.9 | 2349 | 25.1 |  |
|  | Other | 217 | 76.4 | 67 | 23.6 |  |
| ***Country of Residence*** | |  |  |  |  | 0.066 |
|  | England | 6310 | 75.3 | 2070 | 24.7 |  |
|  | Others | 931 | 72.9 | 346 | 27.1 |  |
| ***Residence Area*** | |  |  |  |  | 0.035 |
|  | Urban | 5051 | 74.4 | 1740 | 25.6 |  |
|  | Rural | 2190 | 76.4 | 676 | 23.6 |  |

**Table S2. Distribution of the sample according to confounding variables across trajectories of social mobility**

|  |  | **Social class trajectories** | | | | | | | | |  | |  |
| --- | --- | --- | --- | --- | --- | --- | --- | --- | --- | --- | --- | --- | --- |
|  |  | ***Stable high*** | | ***Upwardly mobile*** | | ***Downwardly mobile*** | | | ***Stable low*** | | | **p-value** | |
|  |  | n | % | n | % | n | % | n | | % |  | |  |
| ***Sex*** | |  |  |  |  |  |  |  | |  | <0.001 | |  |
|  | Male | 1380 | 29.8 | 1192 | 25.7 | 545 | 11.8 | 1516 | | 32.7 |  | |  |
|  | Female | 1669 | 33.2 | 2029 | 40.4 | 357 | 7.1 | 969 | | 19.3 |  | |  |
| ***Ethnicity*** | |  |  |  |  |  |  |  | |  | <0.001 | |  |
|  | White British | 2977 | 31.7 | 3072 | 32.8 | 888 | 9.5 | 2436 | | 26.0 |  | |  |
|  | Other | 72 | 25.3 | 149 | 52.5 | 14 | 4.9 | 49 | | 17.3 |  | |  |
| ***Country of Residence*** | | |  |  |  |  |  |  | |  | 0.013 | |  |
|  | England | 2687 | 32.1 | 2789 | 32.3 | 759 | 9.0 | 2145 | | 25.6 |  | |  |
|  | Others | 362 | 28.4 | 432 | 33.8 | 143 | 11.2 | 340 | | 26.6 |  | |  |
| ***Residence Area*** | |  |  |  |  |  |  |  | |  | <0.001 | |  |
|  | Urban | 2040 | 30.0 | 2382 | 35.1 | 590 | 8.7 | 1779 | | 26.2 |  | |  |
|  | Rural | 1009 | 35.2 | 839 | 29.3 | 312 | 10.9 | 706 | | 24.6 |  | |  |

**Table S3. Association between social class trajectories and self-perceived oral health at 46-48 years of age stratified by gender**

|  |  | Unadjusted model | | Fully adjusted model | |
| --- | --- | --- | --- | --- | --- |
|  |  | OR | 95% CI | OR | 95% CI |
| **Males** | |  |  |  |  |
| Social class trajectories | | |  |  |  |
|  | Stable high (n=3049) | 1.00 | [Reference] | 1.00 | [Reference] |
|  | Upward mobility (n=3221) | 1.36 | [1.14-1.63]** | 1.36 | [1.13-1.63]** |
|  | Downward mobility (n=902) | 1.94 | [1.56-2.42]*** | 1.94 | [1.56-2.41]*** |
|  | Stable low (n=2485) | 2.27 | [1.92-2.68]*** | 2.26 | [1.91-2.66]*** |
| Ethnicity | |  |  |  |  |
|  | White British (n=9373) |  |  | 1.00 | [Reference] |
|  | Other (n=284) |  |  | 0.82 | [0.55-1.22] |
| Country of Residence | |  |  |  |  |
|  | England (n=8380) |  |  | 1.00 | [Reference] |
|  | Others (n=1277) |  |  | 1.11 | [0.92-1.34] |
| Residence Area | |  |  |  |  |
|  | Urban (n=6791) |  |  | 1.00 | [Reference] |
|  | Rural (n=2866) |  |  | 0.92 | [0.80-1.06] |
| **Females** | |  |  |  |  |
| Social class trajectories | | |  |  |  |
|  | Stable high (n=3049) | 1.00 | [Reference] | 1.00 | [Reference] |
|  | Upward mobility (n=3221) | 1.76 | [1.47; 2.10]*** | 1.74 | [1.45; 2.08]*** |
|  | Downward mobility (n=902) | 2.32 | [1.76; 3.06]*** | 2.32 | [1.76; 3.05]*** |
|  | Stable low (n=2485) | 2.66 | [2.18; 3.25]*** | 2.65 | [2.17; 3.23]*** |
| Ethnicity | |  |  |  |  |
|  | White British (n=9373) |  |  | 1.00 | [Reference] |
|  | Other (n=284) |  |  | 1.10 | [0.74; 1.65] |
| Country of Residence | |  |  |  |  |
|  | England (n=8380) |  |  | 1.00 | [Reference] |
|  | Others (n=1277) |  |  | 1.16 | [0.95; 1.44] |
| Residence Area | |  |  |  |  |
|  | Urban (n=6791) |  |  | 1.00 | [Reference] |
|  | Rural (n=2866) |  |  | 0.87 | [0.74; 1.02] |
